# Factors Influencing Decision-Making for Poststroke Paretic Upper Limb Treatment: A Survey of Japanese Physical and Occupational Therapists

**DOI:** 10.1155/2024/1854449

**Published:** 2024-10-07

**Authors:** Koichiro Hirayama, Takashi Takebayashi, Kayoko Takahashi

**Affiliations:** ^1^Graduate School of Rehabilitation, Osaka Metropolitan University, Osaka, Japan; ^2^Graduate School of Medicine, Kitasato University, Kanagawa, Japan

## Abstract

This study investigated the treatment methods used by physical and occupational therapists for poststroke paretic upper limbs and the factors influencing their decision-making processes. For the treatment methods of poststroke paretic upper limbs, the respondents were asked to select the most clinically used treatment according to the severity of the patient's condition. For the factors influencing their decision-making processes, the respondents were asked to indicate each determinant using a 5-point Likert scale (1 = *no influence at all* to 5 = *very strong influence*). Six hundred thirty-eight therapists participated in this study. Exploratory factor analysis was used to assess the validity of the questionnaire. The findings indicated task-specific training (*N* = 333, 52%) as the most popular for mild cases, followed by repetitive facilitative exercise (*n* = 143, 22.3%) for moderate cases and electrical stimulation (*n* = 246, 38.4%) for severe cases. This study revealed that evidence about treatment (very strong: *n* = 171 (27.0%), and strong: *n* = 287 (45.0%)) and patient preferences (very strong: *n* = 203 (31.8%), and strong: *n* = 251 (39.3%)) affected decision-making significantly regarding treatment methods for the poststroke paretic upper limb.

## 1. Introduction

Physical and occupational therapies are vital components of rehabilitation for poststroke paretic upper limbs [1, 2], and treatment interventions, such as constraint-induced movement therapy [3] and mirror therapy [4], have been shown to improve upper limb function in stroke survivors. However, the optimal treatment for poststroke paretic upper limbs remains unclear, and there is no consensus among rehabilitation professionals as the optimal treatment depends on the condition of the stroke patient and the severity of the paralysis [5–7]. This uncertainty is compounded by limited current practices and decision-making process data regarding treating poststroke paretic upper limbs.

Despite the importance of understanding the decision-making process in treating poststroke paretic upper limbs, research on this topic is limited. Latham et al. [8] prospectively monitored the content and intervention techniques of occupational therapy provided by occupational therapists (OTs) and reported that the most time was spent on treatment related to upper extremity control, but the specific interventions were not clear. Connell et al. [9] investigated the factors involved in the decision of physical therapists (PTs) and OTs in the UK to provide treatment for poststroke paretic upper limbs using a cross-sectional study. They reported that the severity of the paretic upper limb, pain, and muscle tone influenced the decision-making process. Furthermore, Springer, Laufer, and Elboim-Gabyzon [10] investigated the factors involved in clinical decision-making when using electrophysical agents by PTs and identified the most influential factors to be equipment availability and patient preferences. Therefore, only a single treatment method has been reported in surveys of clinical decision-making [11, 12].

However, previous studies did not explore decision-making regarding treatment choices for poststroke paretic upper limbs, and clinical decision-making was limited to a single treatment modality.

Evidence-based practice guidelines serve as a resource for PTs and OTs in their treatment decisions [13, 14], and in some studies on decision-making charts based on these guidelines, treatment depended on the decision made among choices [5]. However, research on current treatment therapies with weak evidence of efficacy is insufficient. The decision-making process among therapists varied, with therapists' implicit treatment decisions yielding potentially adverse consequences for patients [15]. Hence, it is imperative to identify the factors influencing a therapist's decision-making process regarding treating poststroke paretic upper limbs.

This study was aimed at investigating the treatment methods for poststroke paretic upper limbs used by PTs and OTs in Japan and the factors influencing their decision-making processes.

## 2. Materials and Methods

### 2.1. Procedures

This study was approved by the Ethics Review Committee of Osaka Metropolitan University (receipt number: 2021-206). In adherence to the “Guidance on Ethical Guidelines for Medical Research Involving Human Subjects” and the Declaration of Helsinki, we ensured that participants provided consent to participate through their responses. The Ethics Review Committee authorized the informed consent procedure and study implementation. At the beginning of the questionnaire, we provided an overview of the study's purpose, content, consent information, and contact details of the researchers. A checkbox was included for participants to indicate their consent, allowing only those who provided consent to proceed with the questionnaire. Additionally, we explained to the participants that they had the option to opt out or withdraw from the study, and in such cases, we committed to communicating with the principal investigator. This study followed the STROBE guidelines (ref: https://www.strobe-statement.org/).

The survey was conducted from August 19 to September 30, 2021. The authors declare no conflicts of interest.

### 2.2. Questionnaire Development

The questionnaire comprised three parts (see Figure S1): “Part 1: demographic characteristics of respondents,” “Part 2: treatment for poststroke paretic upper limb,” and “Part 3: factors that influence PT and OT's decision-making about treatment.” It was developed using questions from studies by Stockley et al. [16] for Parts 1 and 2 and Springer, Laufer, and Elboim-Gabyzon [10] for Part 3. We sought authorization from the primary authors of the referenced studies (Springer, Laufer, and Elboim-Gabyzon [10] and Stockley et al. [16]) to formulate the questionnaire for this study.

The questionnaire used by Springer, Laufer, and Elboim-Gabyzon [10] was developed in consultation with PTs with multiple specialties and focused on factors related to decision-making in selecting a particular treatment. Only an English version of the questionnaire is available; a Japanese version remains undeveloped.

Part 1 of the survey asked about the respondents' occupations, years of experience, working practices, and other demographic characteristics. In Part 2, we presented treatments for the poststroke paretic upper limb, such as constraint-induced movement therapy, mirror therapy, and robotic therapy, and asked the PTs and OTs to select the most clinically used treatments according to the severity of the patient's condition. The severity of the paretic upper limb was classified according to the National Institutes of Health Stroke Scale as follows [17]: mild, 90° shoulder flexion in a seated position (45° in a supine position) for 10 s; moderate, movement possible against gravity but not holding on for at least 10 s; and severe, no movement against gravity but managing to raise the limbs using proximal muscles. The treatments presented in the questionnaire were determined after consultations with six PTs and OTs involved in stroke rehabilitation. Various guidelines and reviews were carefully selected and analyzed [6, 13, 14, 18], along with consultations from numerous extensively experienced field experts engaged in stroke rehabilitation for 5–19 years. The discussions involved six PTs and OTs, including the author. Of the 25 treatments shortlisted, 24 selected treatments were to be presented in the survey after discussion, including mental practice and constraint-induced movement therapy. While treatments, such as “music therapy,” were excluded because their therapeutic focus was not limited to upper limb function, the 24 treatments included rTMS, tDCS, and botulinum toxin injections because the physician made the final treatment decision. However, the decision-making process also heavily involves OTs and PTs.

In Part 3, 13 items influencing PT and OT decisions to treat the paretic upper limb were obtained from previous studies [10]. The 13 items included “learning and training background in training education,” “evidence regarding the efficacy of treatment methods,” “time and ease of treatment,” and “patient preferences and requests.” The respondents were asked to indicate each determinant using a 5-point Likert scale (1 = *no influence at all* to 5 = *very strong influence*).

We followed the procedure for developing the Japanese version of the questionnaire by using the Medical Outcomes Study Short-Form 36-Item Health Survey, repeated forward and reverse translations, and consistency verification [19–21]. Content validation ensured the questionnaire's robustness and validity.

The procedure for preparing this Japanese version is as follows: (1) the principal researcher translated the questionnaire from the original English version into Japanese (forward translation); (2) a bilingual Japanese collaborator, blinded to the original version, translated forward-translated Japanese into English (reverse translation); (3) a different bilingual Japanese collaborator from the bilingual researcher performing the reverse translation compared the original and reverse-translated questionnaire items and examined the consistency using a 10-point scale (0: *no consistency at all* to 10: *strong consistency*); and (4) the principal researcher revised the translation again accordingly. The Japanese version of the questionnaire was completed on attaining a score of 10 for all items (strongly consistent) on repetition.

The completed questionnaire was administered to three PTs and two OTs to confirm the time required to answer the questions and the superficial validity of the questionnaire items. The time required to complete the survey was automatically calculated through Google Forms, confirming that all questions could be answered in less than 15 min. The superficial validity of the questionnaire items was assessed by all PTs and OTs who participated in the pilot test to determine whether any expressions in the questionnaire were difficult to understand. As a result, items such as “technophobia” were reworded as “anxiety about new technology.” Responses from these tests were not included in the study.

### 2.3. Collection of Questionnaire Response Data

The participants of this study were PTs and OTs since they were involved in the rehabilitation for poststroke upper limb paralysis [22]. The survey was distributed from August 19 to September 30. The survey link was shared via social media, and responses were automatically collected through Google Forms. As the responses were obtained through a web-based questionnaire (anonymous), potential respondents self-screened for the following inclusion criteria: (1) they were licensed to practice as PTs or OTs in Japan, (2) they were involved in the treatment of stroke patients, and (3) they could understand written instructions in Japanese. The eligibility for the study was confirmed at the beginning of the questionnaire by asking respondents to select their occupation type. The questionnaire began with an outline of the purpose, content of the study, and the consent to participate. Participants were asked to complete the questionnaire only after fully understanding the study's overview. The sample size *n* and margin of error *E* for this study were calculated using Equations (1)–(3), where *N* is the population size, *r* is the fraction of responses, and *Z*(*c*/100) is the critical value for confidence level *c*.

(1) $$\begin{array}{c} x=Z{\left(\frac{c}{100}\right)}^{2}r\left(100-r\right) \end{array}$$

(2) $$\begin{array}{c} n=\frac{Nx}{\left(\left(N-1\right)E^{2}+x\right)} \end{array}$$

(3) $$\begin{array}{c} E=\text{Sqrt}\left[\frac{\left(N-n\right)x}{n\left(N-1\right)}\right] \end{array}$$

The sample size was calculated using the online Raosoft sample size calculator [23], with a confidence level of 95%, a tolerance of ± 5%, and a response ratio of 0.5, based on a population of approximately 300,000 qualified PTs and OTs in Japan, resulting in a sample size of 384 participants.

### 2.4. Data Analysis

Statistical analyses were performed using the statistical software R 4.2.2. Data on the respondents' demographic characteristics, treatment responses for the paretic upper limb, and 13 items influencing PT and OT decisions were summarized using descriptive statistics such as counts, means, standard deviations (SD), medians, and percentages (percent), as appropriate.

To evaluate the validity of the Japanese version of the questionnaire used in this study, an exploratory factor analysis using Promax rotation was conducted to confirm unidimensionality and to calculate the factor loadings of the questionnaire items. The exploratory factor analysis employed categorical factor analysis with weighted least squares estimation. Generally, factor loadings of less than 0.3 are considered weak, and factor loadings of 0.6 or greater are considered strong. The number of factors was determined based on the criteria of a scree plot, parallel analysis, and factor interpretability based on the item content. The goodness of fit of the factor structure model was determined by examining the Akaike information criterion (AIC), the Bayesian information criterion (BIC), and the root mean square error of approximation (RMSEA). For AIC and BIC, a smaller value indicates a better model, while for RMSEA, a value of 0.10 or higher indicates a poor fit. In addition, the kurtosis and skewness of the responses were assessed, and the mean and SD of the responses were used to determine whether a ceiling or floor effect was present in the question items.

## 3. Results

The survey yielded 640 responses. We analyzed the data from the obtained responses and excluded those (*n* = 2) obtained from individuals other than PTs and OTs. The results of Part 1, “demographic characteristics of respondents,” and Part 2, “treatment methods for poststroke paretic upper limb,” are shown in Tables 1 and 2. The respondents in this study exhibited diverse educational backgrounds, residential locations, and experiences in stroke rehabilitation, implying that the findings can be generalized to the Japanese therapist community. However, a significant majority of respondents (95.2%) are reportedly working in medical facilities, presenting a challenge in extrapolating the findings of the study's results to self-funded (noninsurance covered) and nonprofit organizations.

For mildly paretic upper limbs, 333 (52%) respondents chose task-specific training as their treatment option. In addition, 98 respondents (15.3%) chose constraint-induced movement therapy, and 50 (7.8%) chose repetitive facilitation exercises. For moderately paretic upper limbs, 143 patients (22.3%) opted for repetitive facilitation exercises. Additionally, 122 (19.1%) participants chose task-specific training, and 113 (17.7%) chose electrical stimulation. For severely paretic upper limbs, 246 patients (38.4%) chose electrical stimulation. Additionally, 112 patients (17.7%) elected repetitive facilitation exercises, 63 (10%) selected Bobath therapy, and 62 (9.7%) preferred range-of-motion exercises.

**Figure 1:**
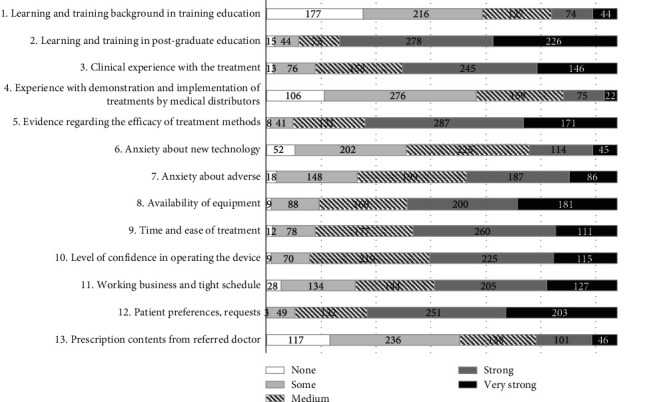
Responses in Part 3. The numbers in the bar chart represent the count for each response option.

The results of Part 3, “factors that influence PT and OT's decision-making about treatment,” are shown in Figure 1. In Part 3, the factors selected as more influential on decision-making were “learning and training in postgraduate education” (very strong: *n* = 226 (35.4%), and very strong: *n* = 278 (43.6%)), “evidence regarding the efficacy of treatment methods” (very strong: *n* = 171 (27.0%), and strong: *n* = 287 (45.0%)), and “patient preferences and requests” (very strong: *n* = 203 (31.8%), and strong: *n* = 251 (39.3%)). The next most frequent responses to the three items were “clinical experience with the treatment” (very strong: *n* = 146 (22.9%), and strong: *n* = 245 (38.4%)), “availability of equipment” (very strong: *n* = 181 (28.4%), and strong: *n* = 200 (31.3%)), and “time and ease of treatment” (very strong: *n* = 111 (17.4%), and strong: *n* = 260 (40.8%)).

Table 3 presents the mean, SD, kurtosis, and skewness of the responses for each factor. In descriptive statistics, skewness and kurtosis in the range of ± 0.5 indicated closeness to the normalcy of the questionnaire item responses to the distribution. The skewness and kurtosis of the responses in this study surpassed 0.5 in either direction, indicating a data bias. However, items with ceiling or floor effects on the mean or SD were absent.

In the exploratory factor analysis, a two- or three-factor structure was indicated based on the scree plot, parallel analysis, and interpretability of item content. The fit indices for the factor structure model were as follows: AIC = 23,066.937, BIC = 23,182.854, and RMSEA = 0.081 for the two-factor structure and AIC = 23,065.125, BIC = 23,185.500, and RMSEA = 0.082 for the three-factor structure. The selection between the two- and three-factor structures was based on model fit and interpretability, favoring the two-factor structure. The factor contribution rates were 19.4% and 0.09% for the first and second factors, respectively.  Table 4 presents the factor loadings of the two-factor structure. Due to factor loadings below 0.30 for Items 12, 13, and 1, unidimensionality confirmation was impossible. Items with factor loadings exceeding 0.6 included “time and ease of treatment,” “availability of equipment,” and “level of confidence in operating the device.”

## 4. Discussion

This study investigated the treatment modalities selected by PTs and OTs in Japan for poststroke paretic upper limbs and the factors influencing the decision-making process.

The task-oriented practice was selected for mild and moderate upper extremity paralysis, and electrical stimulation therapy opted for severe paretic upper limbs. Stockley et al. [16] reported that functional training was the most selected treatment for mild and moderate paretic upper limbs and that ROM training and electrical stimulation therapy were the treatments of choice for severe paretic upper limbs. Task-oriented practice for upper limb motor function has been reported as a component of functional training directed toward clear goals for activities of daily living [24], consistent with the findings of Stockley et al. [16]. Furthermore, concerning treatment choice for severely paretic upper limbs, the study by Stockley et al. [16] showed variability in the treatment used, whereas many therapists in the present study chose electrical stimulation therapy. The stroke treatment guidelines used in Japan recommend task-oriented practice for mild to moderate upper extremity paralysis and electrical stimulation therapy for severe upper extremity paralysis and associated shoulder subluxation. This suggests that the treatments endorsed by the participants in this study align with the stroke treatment guidelines and thus are indicative of their widespread use in clinical practice.

In a study exploring the determinants guiding the treatment decisions of PTs and OTs, equipment availability was identified as the most influential factor [6]. Additionally, Abe, Goh, and Miyoshi [25] reported that past experiences and equipment availability were considered by PT and OT when opting for electrical stimulation therapy. These treatment selection factors partially overlapped with the three influential factors identified in the current investigation. The previous study findings indicate the influence of empirical knowledge and the availability of necessary equipment on the treatment decisions by PT and OT worldwide, as in Japan. Increasing demands on therapists' time highlight the urgent need for the development of tools to streamline the decision-making process with patients, particularly given their hectic schedules. Additionally, there is a pressing requirement for an informational system detailing the multitude of treatments available for upper limb paralysis. However, the questionnaire used in this study may not be generalized to all therapists' decision-making factors. In addition, the questionnaire items in this study only addressed factors related to therapist decision-making and did not consider patient qualities other than the severity of paralysis. Including more detailed characteristics of stroke patients (e.g., stage of recovery and degree of spasticity) may influence the treatment choice for the poststroke paretic upper limb [5].

### 4.1. Limitation

This study had several limitations. First, snowball sampling through social media may have resulted in an imprecise response rate and bias in the participant demographics. In addition, the methodology was designed to obtain a large number of responses and has been widely applied in research [16, 26, 27]. However, the survey method is subject to selection bias. Nevertheless, the total number of responses (640) exceeded the data size required to generate statistics, which may have reduced the sampling error. Therefore, it is possible that the study results were biased toward responses from age groups that are more active on social media or from respondents who have easier access to information. Consequently, the findings may differ from the actual treatment methods and decision-making processes practiced by therapists in clinical settings. Second, the proposed treatment approach may yield different findings compared to previous studies conducted in an unrestricted format. Furthermore, although several therapists with experience in treating stroke decided on the use of the proposed treatment, the respondents may have had different interpretations of the proposed treatment, and the results may not be entirely consistent with clinical practice, thus limiting the scope of the result interpretation. Third, exploratory factor analysis of the questionnaires used in this study revealed low factor contribution rates for Factors 1 and 2. This indicates that the items in the questionnaire are not well related to the latent factors. This suggests that the questionnaire items were not strongly associated with the latent factors, and it is possible that the items did not adequately capture the “decision-making factors for treatment methods” as defined in this study. Therefore, the questionnaire items should be revisited in future studies.

Finally, the results may not be generalizable to other countries or a larger population of therapists because the study was conducted with PTs and OTs in Japan.

## 5. Conclusions

This study highlights the factors influencing PT and OT treatment decisions for poststroke paretic upper limbs, which have rarely been examined in previous studies. These results suggest that PTs and OTs consider multiple factors when selecting treatments, including learning and training in postgraduate education, evidence regarding the efficacy of treatment methods, and patient preferences and requests. Moreover, it emphasized the importance of the environment surrounding the therapist and the PT and OT selection and practice of the latest evidence-based therapy.

## Figures and Tables

**Table 1:** Demographic characteristics of the study sample.

**Characteristic**	**Number (%) or ** **m** **e** **a** **n** ± **SD**
Type of work	
Occupational therapist (OT)	477 (74.5)
Physical therapist (PT)	161 (25.2)
Other	2 (0.3)
Years of experience	8.2 ± 5.6
Educational background	
PhD	19 (3.0)
MSc, MA, or Med	60 (9.4)
BSc	296 (46.3)
Technical school	260 (40.6)
Other	3 (0.4)
Years of therapy for stroke patients	7.4 ± 5.3
Where currently employed	
National health insurance	608 (95.2)
Private medical insurance	5 (0.7)
Nonprofit organization	1 (0.3)
Higher education institutions	5 (0.7)
Other	19 (2.9)
Workplace	
Acute unit	142 (22.2)
General rehabilitation ward	351 (55)
Nursing-care hospital	9 (1.4)
In-home services	59 (9.2)
Facility services	19 (3.0)
Community-based services	2 (0.3)
Local medical institutions	17 (2.7)
Outpatient	13 (2.0)
Other	26 (4.0)

**Table 2:** Selection of treatment for each severity number (%).

**Treatment**	**Mild**	**Moderate**	**Severe**
Bobath therapy	36 (5.6)	62 (9.7)	63 (9.8)
Mental practice	3 (0.5)	2 (0.3)	6 (0.9)
Mirror therapy	2 (0.3)	4 (0.6)	13 (2.0)
Robotic therapy	8 (1.2)	27 (4.2)	28 (4.4)
Stretching	1 (0.2)	2 (0.3)	6 (0.9)
Virtual reality (VR)	0 (0)	0 (0)	1 (0.2)
Bilateral arm training	33 (5.2)	32 (5.0)	28 (4.4)
Repetitive transcranial magnetic stimulation (rTMS)	0 (0)	2 (0.3)	3 (0.5)
Transcranial direct current stimulation (tDCS)	0 (0)	0 (0)	0 (0)
Invasive motor cortex stimulation (MCS)	0 (0)	0 (0)	0 (0)
Constraint-induced movement therapy (CIMT)	98 (15.3)	37 (5.8)	3 (0.5)
Electrical stimulation	12 (1.9)	113 (17.7)	246 (38.4)
Task-specific training	333 (52.0)	122 (19.1)	9 (1.4)
Botulinum toxin injection	0 (0)	0 (0)	1 (0.2)
Biofeedback	0 (0)	3 (0.5)	3 (0.5)
Strength training	34 (5.3)	45 (7.0)	8 (1.2)
Action observation	5 (0.8)	4 (0.6)	1 (0.2)
Hands-on therapy	8 (1.2)	22 (3.4)	14 (2.2)
Positioning	0 (0)	0 (0)	5 (0.8)
Range-of-motion exercises	14 (2.2)	12 (1.9)	62 (9.7)
Wearing an arm sling	1 (0.2)	4 (0.6)	17 (2.7)
Repetitive facilitative exercise	50 (7.8)	143 (22.3)	112 (17.5)
Orthosis in hemiparetic upper extremity	0 (0)	2 (0.3)	9 (1.4)
Telerehabilitation	0 (0)	0 (0)	0 (0)

*Note:* Hands-on therapy: exercises in which some or all of the joints of a patient's arm or hand are manually assisted to maintain joint and soft tissue mobility. Severity classification based on the National Institutes of Health Stroke Scale (mild–severe): mild: able to hold 90° of shoulder flexion in a sitting position (45° in a supine position) for 10 s; moderate: moves against gravity but cannot hold it for 10 s; and severe: no movement against gravity but can manage to raise using proximal muscles.

**Table 3:** Items influencing treatment choices for the poststroke paretic upper limb.

**Item no.**	**Item**	**Mean ± standard deviation**	**Skewness**	**Kurtosis**
1	Learning and training background in training education	2.4 ± 1.2	0.6539	−0.4677
2	Learning and training in postgraduate education	4.0 ± 1.0	−1.1013	0.9261
3	Clinical experience with the treatment	3.7 ± 1.0	−0.4774	−0.4223
4	Experience with demonstration and implementation of treatments by medical distributors	2.4 ± 1.0	0.5773	−0.1229
5	Evidence regarding the efficacy of treatment methods	3.9 ± 0.9	−0.6999	0.2489
6	Anxiety about new technology	2.8 ± 1.0	0.2645	−0.4543
7	Anxiety about adverse effects	3.3 ± 1.1	−0.0117	−0.8184
8	Availability of equipment	3.7 ± 1.1	−0.3826	−0.8108
9	Time and ease of treatment	3.6 ± 1.0	−0.4087	−0.3474
10	Level of confidence in operating the device	3.6 ± 1.1	−0.2160	−0.4670
11	Working business and tight schedule	3.4 ± 1.6	−0.2619	−0.9223
12	Patient preferences and requests	3.9 ± 1.0	−0.5944	−0.3474
13	Prescription contents from a referred doctor	2.6 ± 1.5	0.4753	−0.6500

**Table 4:** Factor loadings for each item in the two-factor model.

**Item no.**	**Item**	**Factor**	
		**1**	**2**
9	Time and ease of treatment	**0.639**	−0.098
8	Availability of equipment	**0.639**	−0.138
10	Level of confidence in operating the device	**0.603**	−0.075
11	Working business and tight schedule	**0.527**	−0.030
6	Anxiety about new technology	**0.514**	−0.010
7	Anxiety about adverse effects	**0.459**	0.140
3	Clinical experience with the treatment	0.396	−0.014
2	Learning and training in postgraduate education	0.392	−0.024
5	Evidence regarding the efficacy of treatment methods	0.330	0.091
4	Experience with demonstration and implementation of treatments by medical distributors	0.307	0.078
12	Patient preferences and requests	0.271	0.198
13	Prescription contents from a referred doctor	−0.348	0.135
1	Learning and training background in training education	0.186	0.218

*Note:* Factor loadings of ± 0.4 or greater are shown in bold.

## Data Availability

The participants of this study did not give written consent for their data to be shared publicly, so due to the sensitive nature of the research, supporting data is not available.

## Supporting Information

Supporting Information Additional supporting information can be found online in the Supporting Information section. Figure S1. The questionnaire used in this study was developed based on previous research. To ensure the accuracy of the content and transparency of the methodology in our paper, this supporting information provides the translated English version of the original Japanese questionnaire. The translation was carefully conducted to accurately convey the intent of the questions.
