# Supplementary material for: Factors Influencing Decision-Making for Poststroke Paretic Upper Limb Treatment: A Survey of Japanese Physical and Occupational Therapists
Source: Occup Ther Int. 2024 Oct 7;2024:1854449. doi: 10.1155/2024/1854449 (PMC11473170; doi:10.1155/2024/1854449)
Supplement: Supporting Information — Additional supporting information can be found online in the Supporting Information section. Figure S1. The questionnaire used in this study was developed based on previous research. To ensure the accuracy of the content and transparency of the methodology in our paper, this supporting information provides the translated English version of the original Japanese questionnaire. The translation was carefully conducted to accurately convey the intent of the questions. [file 1854449.f1.docx]

**Questionnaire on Therapy Options for Post-Stroke Paretic Upper Limb**

**【Aim of this Study】**

Recently, Evidence-Based Medicine (EBM) has become widespread in the rehabilitation field, and there has been an increasing movement toward the provision of medical care that integrates the effectiveness of treatments proven through clinical research, the wishes of patients, and the expertise of medical professionals.

The efficacy of treatment for upper limb motor paresis after stroke has been verified by numerous studies. The number of studies is much larger than in other areas of rehabilitation research. On the other hand, regarding therapists who provide therapy, previous studies have reported that therapists implicitly determine the therapy and that patients were not provided with enough information to participate in the decision-making process. In response to these issues, previous studies have investigated the therapies that therapists working with stroke patients routinely provide and the factors that led them to choose those therapies (Rachel et al., 2019; Shmuel et al., 2015). However, there are no studies that have conducted a nationwide survey of therapists' treatment choices for upper limb motor paresis after stroke in Japan, and what factors influence their decision-making.

Therefore, the aim of this study was to conduct a nationwide survey of occupational therapists and physical therapists in Japan regarding therapy choices for patients with post-stroke paretic upper limb. This study will not only clarify what kind of treatment is being offered to stroke patients in the rehabilitation field in Japan but will also help support decision-making between patients and therapists regarding therapy.

**【Methods and Duration】**

This study will be cross-sectional survey research using a web-based questionnaire (unnamed). The overall period of the study will be from the date of approval by the Research Ethics Committee of the research institution to which the principal investigator belongs until March 31, 2026.

**【Subjects】**

Physical and occupational therapists working in Japan.

**【Handling of personal information】**

The survey will be conducted without names. The names of respondents will not be collected, and the information provided will be carefully managed to ensure that it is not lost or leaked to outside parties, thereby protecting privacy and personal information.

**【Methods of storage and disposal of information】**

The data from the questionnaire will be stored on a USB memory stick in a lockable locker in the Rehabilitation Department of Kishiwada Rehabilitation Hospital, where the principal investigator belongs, for five years after the completion of the study. After the completion of the research, the data in the USB flash drive will be destroyed using deletion software on the computer.

**【Consent and Withdrawal for Cooperation in this Study】**

Consent for participation in this research was obtained based on the following four points: (1) this research does not involve any invasive procedures (except for minor invasions), (2) simplification of the informed consent procedure will not be detrimental to the research subjects, (3) it is difficult to conduct this research without simplification of the informed consent procedure, and (4) the study is considered to be of high social importance. Therefore, following the "Ethical Guidelines for Medical Research Involving Human Subjects," we will simplify the usual informed consent process, and consent will be obtained by answering the questionnaire on the website. You may interrupt the questionnaire in the middle of the survey. However, when withdrawing your consent, please understand that you cannot withdraw your participation in the research if the research results have already been published in a conference presentation or academic paper, or if you have already responded to an unsigned questionnaire.

**【Predicted Benefits and Disbenefits】**

There are no direct benefits to be derived from respondents' participation in this study. Please understand that this type of study is conducted for medical development. In addition, there may be time and physical burdens on respondents due to their responses to questionnaires.

**【Methods of Disclosure of Information on this Study】**

After the completion of this study, the principal researcher will summarize the results obtained from you and release them to the public through conference reports, submission to academic journals, and other means.

【Contact information for inquiries】

If you have any questions about this study, please contact the principal researcher.

The principal researcher： Kouichiro Hirayama

Graduate School of Comprehensive Rehabilitation, Osaka Prefecture University, Osaka, Japan

Department of Rehabilitation, Eishinkai Kishiwada Rehabilitation Hospital, Osaka, Japan

E-mail：akfg96@icloud.com

Co-researchers： Takashi Takebayashi

Graduate School of Comprehensive Rehabilitation, Osaka Prefecture University, Osaka, Japan.

E-mail：mf701015@edu.osakafu-u.ac.jp

If you are willing to cooperate with this study, please answer the questionnaire after selecting "I agree".

Please mark only one.

- I agree

**Part 1：Demographic Characteristics**

1. Are you a physical or occupational therapist working in Japan?

Please mark only one.

- Physical therapist
- Occupational therapist
- Other：

1. How many years have been qualified?

Please enter a number.

1. What is your highest academic qualification?

Please mark only one.

- PhD
- MSc, MA, or MEd
- BSc
- Technical School
- Other：

1. How many years have you worked with people who have had a stroke?

Please enter a number.

1. Do you currently work clinically with stroke survivors with upper limb deficits at any stage of their rehabilitation?

Please mark only one.

- Yes
- No

1. Where are you currently employed?

Please mark only one.

- National health insurance
- Private medical insurance
- Non-profit organization
- Higher education institutions
- Other：

1. Please indicate the prefecture where you work.

Please mark only one.

- Hokkaido
- Aomori
- Iwate
- Miyagi
- Akita
- Yamagata
- Fukushima
- Ibaraki
- Tochigi
- Gunma
- Saitama
- Chiba
- Tokyo
- Kanagawa
- Niigata
- Toyama
- Ishikawa
- Fukui
- Yamanashi
- Nagano
- Gifu
- Shizuoka
- Aichi
- Mie
- Shiga
- Kyoto
- Osaka
- Hyogo
- Nara
- Wakayama
- Tottori
- Shimane
- Okayama
- Hiroshima
- Yamaguchi
- Tokushima
- Kagawa
- Ehime
- Kochi
- Fukuoka
- Saga
- Nagasaki
- Kumamoto
- Oita
- Miyazaki
- Kagoshima
- Okinawa

1. Where are you currently employed?

Please mark only one.

- Acute unit
- General rehabilitation ward
- Nursing-care hospital
- In-home services
- Facility services
- Community-based services
- Local medical institutions
- Outpatient
- Other：

**Part 2：Treatment for post-stroke paretic upper limb**

In this section, for each severity of the paretic upper limb based on the National Institutes of Health Stroke Scale (NIHSS), please select one of the following treatments that you would most likely use in your clinical practice.

- Mild: able to hold 90° of shoulder flexion in a sitting position (45° in a supine position) for 10 seconds.

Please mark only one.

- Bobath therapy
- Mental practice
- Mirror therapy
- Robotics therapy
- Stretching
- Virtual reality
- Bilateral arm training
- Repetitive transcranial magnetic stimulation
- Transcranial direct current stimulation
- Invasive motor cortex stimulation
- Constraint-induced movement therapy
- Electrical stimulation
- Task-specific training
- Botulinum toxin injection
- Biofeedback
- Strength training
- Action observation
- Hands-on therapy
- Positioning
- Range of motion exercises
- Wearing an arm sling
- Repetitive facilitative exercise
- Orthosis in hemiparetic upper extremity
- Telerehabilitation
- Moderate: moves against gravity but unable to hold for 10 seconds.

Please mark only one.

- Bobath therapy
- Mental practice
- Mirror therapy
- Robotics therapy
- Stretching
- Virtual reality
- Bilateral arm training
- Repetitive transcranial magnetic stimulation
- Transcranial direct current stimulation
- Invasive motor cortex stimulation
- Constraint-induced movement therapy
- Electrical stimulation
- Task-specific training
- Botulinum toxin injection
- Biofeedback
- Strength training
- Action observation
- Hands-on therapy
- Positioning
- Range of motion exercises
- Wearing an arm sling
- Repetitive facilitative exercise
- Orthosis in hemiparetic upper extremity
- Telerehabilitation
- Severe: no movement against gravity (can raise using proximal muscles).

Please mark only one.

- Bobath therapy
- Mental practice
- Mirror therapy
- Robotics therapy
- Stretching
- Virtual reality
- Bilateral arm training
- Repetitive transcranial magnetic stimulation
- Transcranial direct current stimulation
- Invasive motor cortex stimulation
- Constraint-induced movement therapy
- Electrical stimulation
- Task-specific training
- Botulinum toxin injection
- Biofeedback
- Strength training
- Action observation
- Hands-on therapy
- Positioning
- Range of motion exercises
- Wearing an arm sling
- Repetitive facilitative exercise
- Orthosis in hemiparetic upper extremity
- Telerehabilitation

**Part 3： Factors that influence Physical and occupational therapists' Decision-making about treatment**

In this section, please choose only one item that best describes the degree to which each of the items (decision factors) affects your choice of therapy, ranging from 1: not at all to 5: very strongly.

1. Learning and training background in training education.

- none
- some
- medium
- strong
- very strong

1. Learning and training in post-graduate education.

- none
- some
- medium
- strong
- very strong

1. Clinical experience with the treatment

- none
- some
- medium
- strong
- very strong

1. Experience with demonstration and implementation of treatments by medical distributors.

- none
- some
- medium
- strong
- very strong

1. Evidence regarding the efficacy of treatment methods.

- none
- some
- medium
- strong
- very strong

1. Anxiety about new technology.

- none
- some
- medium
- strong
- very strong

1. Anxiety about adverse effects.

- none
- some
- medium
- strong
- very strong

1. Availability of equipment.

- none
- some
- medium
- strong
- very strong

1. Time and ease of treatment.

- none
- some
- medium
- strong
- very strong

1. Level of confidence in operating the device.

- none
- some
- medium
- strong
- very strong

1. Working business and tight schedule

- none
- some
- medium
- strong
- very strong

1. Patient preferences, requests.

- none
- some
- medium
- strong
- very strong

1. Prescription contents from referred doctor.

- none
- some
- medium
- strong
- very strong

Thank you for taking the time to complete the survey.
